# Fungal Disease and the Developing Story of Bat White-nose Syndrome

**DOI:** 10.1371/journal.ppat.1002779

**Published:** 2012-07-19

**Authors:** David S. Blehert

**Affiliations:** US Geological Survey – National Wildlife Health Center, Madison, Wisconsin, United States of America; Duke University Medical Center, United States of America

Two recently emerged cutaneous fungal diseases of wildlife, bat white-nose syndrome (WNS) [1] and amphibian chytridiomycosis [2], have devastated affected populations. Fungal diseases are gaining recognition as significant causes of morbidity and mortality to plants, animals, and humans [3], yet fewer than 10% of fungal species are known [4]. Furthermore, limited antifungal therapeutic drugs are available, antifungal therapeutics often have associated toxicity, and there are no approved antifungal vaccines. The unexpected emergence of WNS, the rapidity with which it has spread, and its unprecedented severity demonstrate both the impacts of novel fungal disease upon naïve host populations and challenges to effective management of such diseases.

## When Was WNS Discovered and What Is the Cause?

The first evidence suggestive of WNS in North America is a photograph of hibernating bats with an unusual white substance on their muzzles taken in February 2006, at a cave in east central New York. Since then, WNS has spread to 19 US states and four Canadian provinces (see http://www.fws.gov/whitenosesyndrome/maps/WNSMAP04-27-12_300dpi.jpg) and is estimated to have killed over 5 million insectivorous bats (see http://www.fws.gov/whitenosesyndrome/pdf/WNS_Mortality_2012_NR_FINAL.pdf). A recent study predicted the little brown bat (*Myotis lucifugus*), a species particularly hard-hit by WNS and once the most common bat species in the northeastern US, may be regionally extirpated by the year 2026 as a result of this disease [5]. Epizootic disease on the order of WNS is unprecedented among wild mammals.

**Figure 1:**
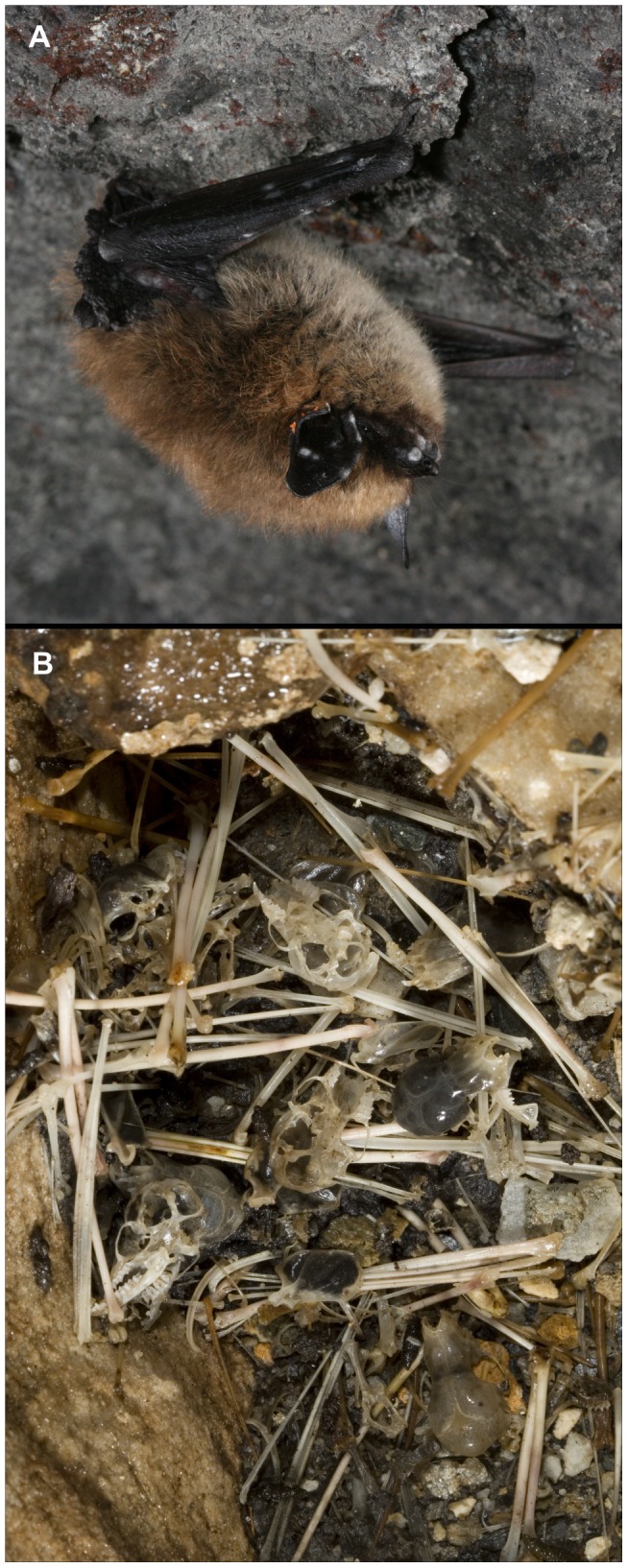
Images documenting clinical signs of white-nose syndrome (WNS) and devastating effects of the disease upon a wild bat population. (A) Eastern small-footed bat (*Myotis leibii*) with WNS photographed in April 2009, in Graphite Mine, New York, USA. Note characteristic white and gray fungal growth on the bat's muzzle, ear, and wing. (B) Bat skeletal remains on the floor of Aeolus Cave, Vermont, USA, photographed in 2010, approximately two years after white-nose syndrome was first identified at this site. Photo credit: Alan Hicks.

Biologists first observed unusual mortality and other clinical signs suggestive of WNS (Figure 1) in March 2007, at five underground bat hibernation sites (hibernacula) near Albany, New York. A multi-institutional disease investigation was initiated in January, 2008. The gross clinical presentation of WNS, a white substance on bats' muzzles, ears, and wings, suggested a fungal cause. A key finding was made when microscopy revealed that bats were colonized by a fungus with unique curve-shaped conidia. In parallel, skin samples from infected bats cultured at 7°C, a temperature consistent with the body temperature of hibernating bats, consistently yielded isolates of this same fungus [1], [6], a previously unknown species that was subsequently named *Geomyces destructans* [7].

Infection trials have since demonstrated that *G. destructans* is the sole causative agent of WNS [8], [9]. As a psychrophile, growth of *G. destructans* is restricted to temperatures below approximately 20°C [1], [7], making WNS an unusual disease among “warm blooded” mammals. Bats infected by *G. destructans* only develop WNS during hibernation when they dramatically reduce their core body temperature to a level conducive to proliferation of the fungus. Because of this temperature restriction, *G. destructans* is not known to be a pathogen of humans or domestic animals, and WNS is widely perceived strictly as a wildlife conservation problem, not as a threat to public health or agricultural production. However, while insectivorous bats lack the readily definable market value of domestic animals, they have great value both as critical components of ecosystems and for the ecological services they provide. A recent analysis estimated that insect suppression services provided by bats to commercial agriculture in the continental US has an average value of $22.9 billion per year [10].

## Is *G. destructans* Related to Other Fungal Pathogens?

Ribosomal RNA gene region sequence analyses place the WNS pathogen in the anamorphic genus *Geomyces*, which consists predominately of cold-tolerant environmental saprobes [11]. Within the phylum Ascomycota, *Geomyces* belongs to the class Leotiomycetes, a group of fungi that includes several important plant pathogens (e.g., *Botrytis cinerea* and *Schlerotinia sclerotiorum*) but that is not recognized to include major pathogens of animals [12]. With the exception of a few reports of *G. pannorum* acting as a dermatophyte [13], *Geomyces* spp. are not commonly recognized as animal pathogens. Work is ongoing to identify characteristics that differentiate *G. destructans* from its non-pathogenic relatives.

## Has *G. destructans* Been Found Beyond the Boundaries of North America?

Following the initial description of *G. destructans* in North America, investigators found the same fungus on hibernating bats in at least 12 European countries but without associated unusual mortality among infected animals [14]. It was then shown that isolates of *G. destructans* from North America were of a single clonal genotype [15], and an experimental study demonstrated that a European isolate of *G. destructans* was lethal to little brown bats, a North American bat species that does not exist in Europe [9]. Together, these findings support the hypothesis that the fungus was introduced from a foreign source to North America where it is acting as an emergent pathogen among a naïve population of hosts. Although differences in WNS lethality among bats of North America and Europe are still not understood, they likely stem from intercontinental differences in bat population dynamics, host species, and/or environmental conditions within hibernacula. Planned laboratory infectivity studies with a European bat species may yield additional insights.

## Why Is *G. destructans* Pathogenic to Hibernating Bats?

While pathogenesis of *G. destructans* is not yet fully elucidated, an unrelated fungal skin infection of wildlife, amphibian chytridiomycosis caused by the fungus *Batrachochytrium dendrobatidis* [16], may provide important clues toward developing a more complete understanding of WNS. Skin of amphibians plays a vital role in maintaining osmotic homeostasis, and infection by *B. dendrobatidis* inhibits electrolyte transport across the epidermis of infected animals, disrupting plasma sodium and potassium concentrations and causing cardiac arrest and death [17]. Unlike *B. dendrobatidis* and other fungal dermatophytes that superficially colonize epidermis, nails, and hair, *G. destructans* is an aggressive fungal pathogen that penetrates the epidermis and invades underlying connective tissues [18]. Pathology analyses have shown the primary site for infection by *G. destructans* is the skin of bats' wings [18], [19]. In addition to its unique role in mammalian flight, bat wing skin also serves a critical role in maintaining physiological homeostasis. Intact bat wing skin prevents evaporative water loss, helps to release carbon dioxide through passive cutaneous exchange, aids in regulation of blood pressure, and supports thermoregulatory processes [19]. The importance of these physiological functions may be further elevated during hibernation, the exclusive time when bats are vulnerable to infection by *G. destructans* and a physiological state that may render them immunologically or otherwise unable to counteract both fungal infection and its sequelae. Additionally, infection of bats by *G. destructans* has been shown to cause increased arousal from torpor during hibernation, which likely contributes to depletion of energy reserves and death [9], [20]. A comprehensive understanding of disruptions to the delicate physiology of hibernating bats, either caused directly by the invasive skin infection that characterizes WNS and/or as mediated by yet uncharacterized fungal effector proteins, will likely be key to understanding this deadly disease.

## What Can We Learn from the Discovery of WNS?

Recent applications of culture-independent microbial discovery techniques have yielded extraordinary advances in pathogen discovery [21]. However, the story of the discovery of *G. destructans* demonstrates that traditional culture- and histopathology-based laboratory methods remain highly relevant in disease investigation. For a cutaneous fungal disease such as WNS, the high diversity of fungi found in environments occupied by hibernating bats [22] and the non-sterile nature of skin makes the identification of a single clinically relevant microorganism difficult. Additionally, classification of the agent causative of WNS among Leotiomycetes, fungi not previously recognized as primary pathogens of animals [12], further complicates differentiation of a fungal pathogen from common environmental microflora. In the case of WNS, morphological characterization of the infectious agent in laboratory culture and on the skin of infected bats by both direct microscopy and histopathology readily provided the ability to make the original connection between *G. destructans* and WNS [1], [6], [18]. Definitive identification of *G. destructans* as the causative agent of WNS was ultimately determined through fulfillment of Koch's Postulates using a model system involving hibernating little brown bats [8]. Careful field observations, detailed pathology analyses, and controlled experimental confirmations bringing together scientists from multiple disciplines including microbiology, pathology, and ecology will continue to remain important in determining etiologies of novel fungal diseases.

## Conclusions

Infectious diseases occur when a pathogen is introduced into a population of susceptible hosts under appropriate environmental conditions. In the case of WNS, the pathogen is *G. destructans* [8], the hosts are hibernating bats [1], and the environments that promote development of disease are cold underground hibernacula that bats occupy during winter. Unlike pathogenic microbes such as viruses that require host species for their survival, fungal pathogens can survive in the environment in the absence of hosts, providing them with the unique potential to extirpate host populations [3]. Thus, WNS presents a dire threat to populations of insectivorous hibernating bats in North America. However, much is known about the physiology of bat host species [19], and since the first published description of WNS [1], we have isolated and identified the pathogen [7] and developed model systems to study host–pathogen aspects of WNS in the laboratory [8], affording opportunities to fully define mechanisms of WNS pathogenesis. To date, efforts to manage the spread of WNS have focused on implementation of universal precautions, including restricting access of humans to sensitive bat hibernation sites and decontaminating equipment and clothing when sites are accessed for disease surveillance, research, or recreational purposes. Despite the considerable challenge of managing infectious disease in free-ranging wildlife while avoiding unintended adverse consequences, additional research aimed at increasing our understanding of the ecology of WNS in bats and their environment continues to offer the greatest potential for identifying novel strategies to mitigate the effects of this unprecedented disease.
